# The complete mitochondrial genome of Indo-Pacific soft coral *Sinularia acuta* Manuputty and van Ofwegen, 2007 (Octocorallia: Alcyonacea)

**DOI:** 10.1080/23802359.2023.2184658

**Published:** 2023-03-08

**Authors:** Chaojie Yang, Farnaz Mahmoudi Shikhsarmast, Chunzheng Fu, Chun-Yang Shen

**Affiliations:** a Key Laboratory of Utilization and Conservation for Tropical Marine Bioresources, Hainan Tropical Ocean University, Sanya, China; b Institute of Sericulture, Chengde Medical University, Chengde, Hebei, China; c Department of Biology, Chengde Medical University, Chengde, Hebei, China

**Keywords:** *Sinularia acuta*, soft coral, *Sinularia*, mitogenome, phylogenetic status

## Abstract

The complete mitochondrial genome (mitogenome) of the soft coral *Sinularia acuta* Manuputty and van Ofwegen, 2007 was sequenced and annotated using Illumina next-generation sequencing (NGS). The mitogenome of *S. acuta* was 18,730 bp in length and consisted of 14 protein-coding genes (PCGs), two ribosomal RNA genes (rRNA), and only one transfer RNA gene (*tRNA-Met*). The base composition was 30.18% A, 16.46% C, 19.35% G, and 34.00% T, with a total A + T content of 64.19%. The phylogenetic analysis demonstrated a close evolutionary relationship among *Sinularia acuta*, *Sinularia penghuensis*, and *Sinularia maxima*.

## Introduction

The soft coral genus *Sinularia* May 1898, also called short fingered soft coral, belongs to Alcyonacea: Alcyoniidae. The species of *Sinularia* are distributed in extensive environmental fields in Indo-Pacific open water, from shallow to deep reefs. They have various growth patterns and different sizes of colonies (Fabricius and Alderslade 2001). *Sinularia acuta* Manuputty and van Ofwegen, 2007 was described by Manuputty and van Ofwegen from Moluccas (Indonesia) in 2007. The South China Sea is one of the habitats of this species in Southeast Asia (Benayahu et al. 2012, 2018).

The soft coral *Sinularia*, with more than 170 described species, is a large genus in Octocorallia; therefore, its mitochondrial genome information can draw a clear evolutionary relationship among species, but few mitochondrial genomes have been sequenced (Asem et al. 2019; Chen et al. 2019; Shen et al. 2021). In this study, we sequenced and annotated the complete mitochondrial genome of *S. acuta* (GenBank no. MW987591), and a phylogenetic analysis was performed to investigate its relationships with other *Sinularia* species.

## Materials and methods

**Figure 1.**
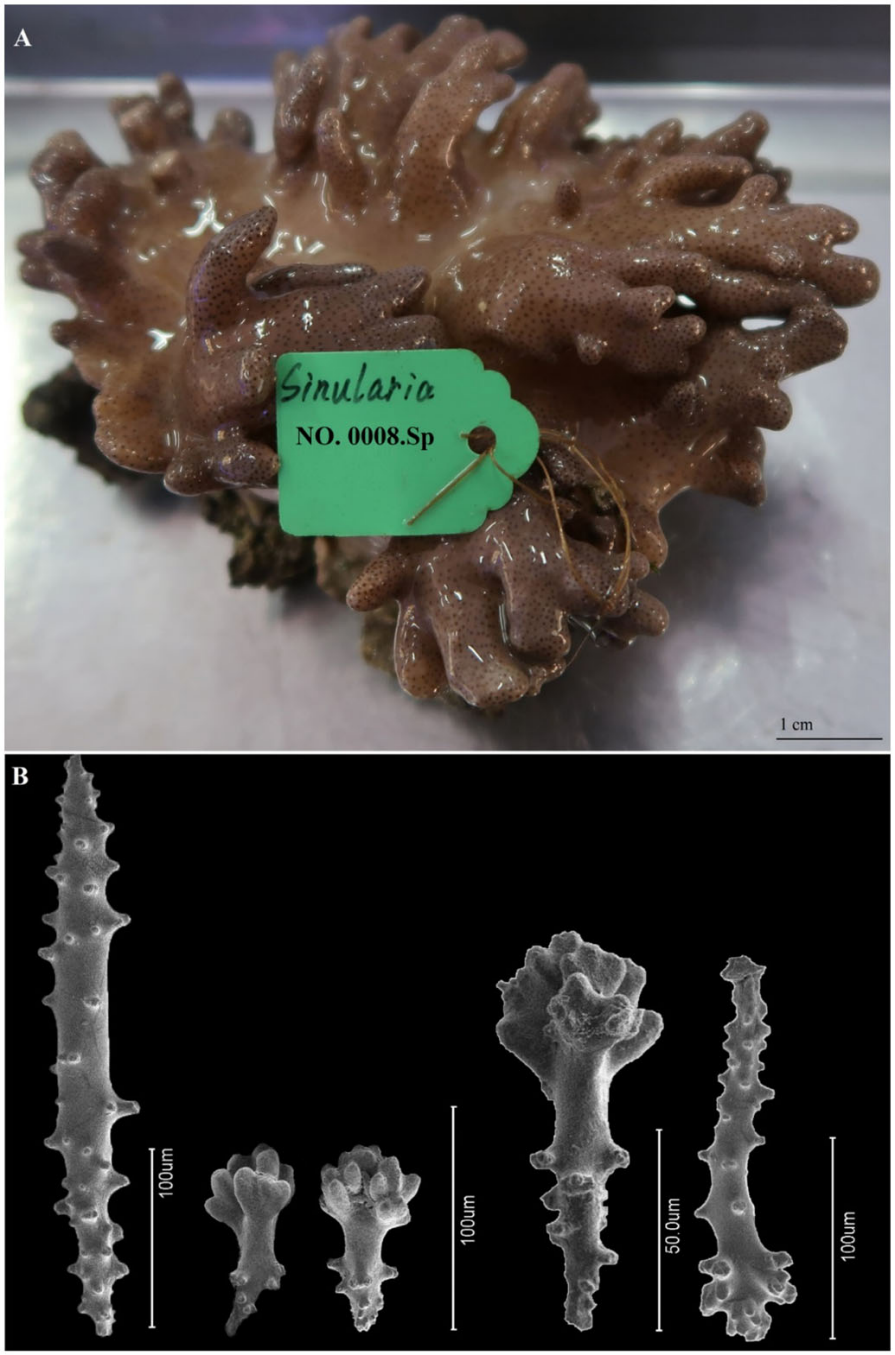
(A) Image of the live individual reference specimen of *Sinularia acuta* Manuputty and van Ofwegen, 2007 from West Island (Sanya, Hainan Province, China; 18° 14′ 5.93″ N, 109° 22′ 46.46″ E). (B) SEM morphology of sclerites from the colony polyps and surface of *S. acuta* (photos by Chaojie Yanga).

To collect the sample, permission was obtained from the Hainan Province government (Department of Science and Technology, reference number ZDYF2019154). Additionally, the specimen collection and experimental protocol of this study were approved by the Ethical Review Department of Science and Technology of Hainan Province (China) (reference number ZDKJ2019011-03-02). A live specimen of *S. acuta* was collected in West Island (Sanya, Hainan Province, China; 18° 14′ 5.93″ N, 109° 22′ 46.46″ E), and its taxonomic status was confirmed based on the morphology of sclerites following McFadden et al. (2009). The specimen and its DNA were deposited at the Hainan Tropical Ocean University Museum of Zoology (specimen voucher number: 0008-Sp; DNA ID number: 0008-D; Chaojie Yang: duanduan1986@outlook.com). Figure 1 shows the specimen reference image and morphology of sclerites.

**Figure 2.**
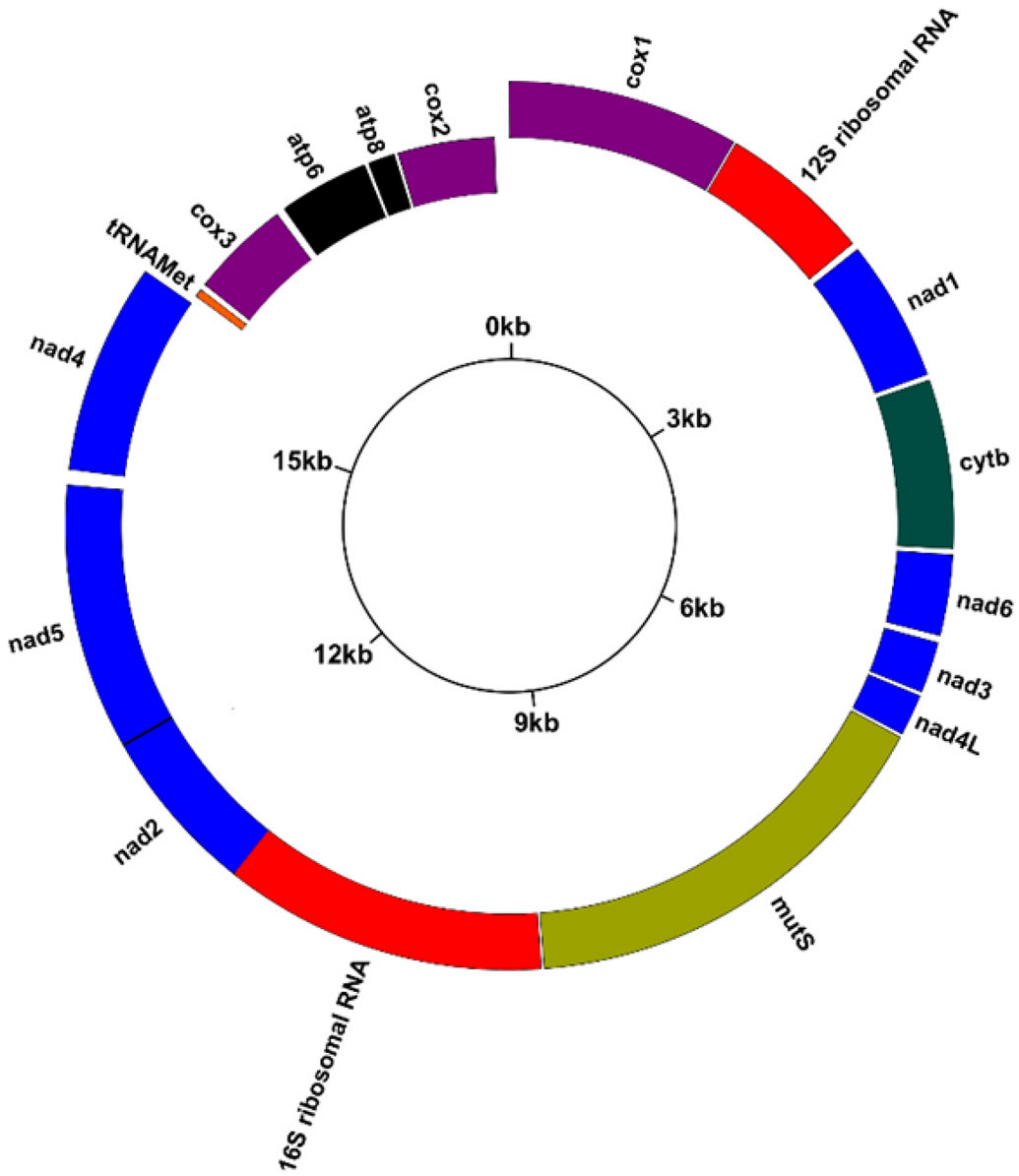
Circular map of the complete mitochondrial genome (GenBank no. MW987591) of the short fingered soft coral *S. acuta*. Outside position: heavy strand, inside position: light strand.

Total genomic DNA was extracted from 30 mg of tissue using Genomic Animal DNA Isolation (Sangon Biotech Co., Kit NO. B518221, Shanghai, China) (Asem et al. 2021). A paired-end genomic library was arranged (10 GB; two pair reads: 150 bp) utilizing the Illumina HiSeq X-ten. FastQC software was used to control the quality of the reads (Andrews 2010). Information on classification of raw reads and quality score distribution along reads were shown in Figure S1. The complete mitochondrial genome of *Sinularia ceramensis* (NC_044122) was chosen to perform the de novo assemblies by Geneious v.9.1 (Kearse et al. 2012). The GenomeVx online platform (http://wolfe.ucd.ie/GenomeVx/) was used to draw the mitogenomic circular map of *S. acuta* (Figure 2). The ARWEN online platform was used to determine the position of *tRNA-Met* (http://130.235.46.10/ARWEN/). BioEdit software was employed to annotate the positions of protein-coding genes and ribosomal RNA genes using the reference mitogenome (Hall 1999). Additionally, the position and orientation of all PCGs were reconsidered by translation of amino acid sequences (ExPASy online tool: https://web.expasy.org/translate/) and the position of both the start codon and stop codon in each PCG. Maximum likelihood (ML) phylogenetic analysis was performed utilizing the software MEGA X (Kumar et al. 2018) with 1000 bootstrap replicates and a GTR model, which was determined by jmodeltest v.2.1.10 (Darriba et al. 2012). The concatenated sequences of 14 PCGs were used to draw a phylogenetic tree.

## Results

Mean depth information for the assembly of complete mitochondrial genome of *S. acuta* was represented in Figure S2. The mitogenome of *S. acuta* was 18,730 bp in length. The overall base compositions were 30.18% A, 16.46% C, 19.35% G, and 34.00% T, with a total A + T content of 64.19%. Similar to the other published Alcyoniidae mitogenomes, 17 genes were detected in the mitogenome of *S. acuta*, including 14 PCGs, two rRNAs, and one tRNA (*tRNA-Met*). Ten PCGs (*MutS*, *cox1*, *cytb*, *nad1*, *nad2*, *nad3*, *nad4*, *nad4L*, *nad5*, and *nad6*) and two rRNAs (*12S* and *16S*) were encoded on the heavy chain. The other four PCGs (*cox2*, *cox3*, *atp6*, and *atp8*) and *tRNA-Met* were located on the light chain.

All PCGs used ATG as the initiation codon. Five PCGs, including *nad4L*, *MutS*, *nad4*, *atp6*, and *atp8*, had TAA as a stop codon, while *cox1* consisted of a non-complete codon (T−). There were 16 intergenic spacers in the present mitogenome. The major non-coding region was between *cox2* and *cox1* (112 bp), and a 13 bp overlap was detected between *nad2* and *nad5*.

## Discussion

**Figure 3.**
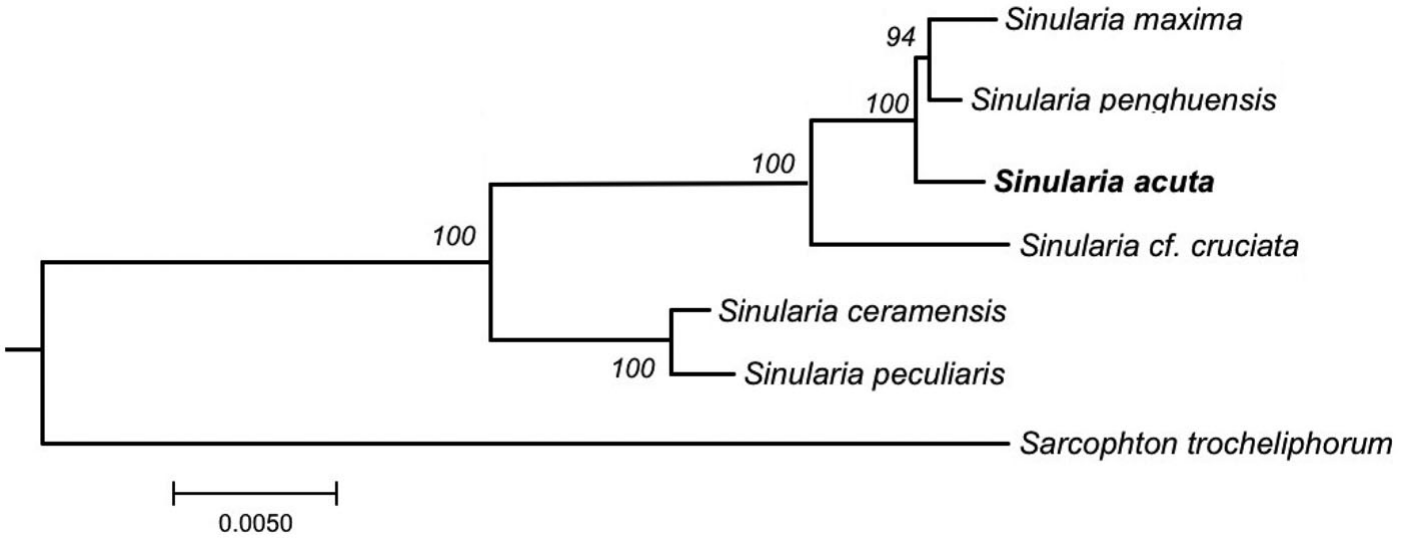
Phylogenetic tree showing the relationship of the genus *Sinularia* based on the concatenated nucleotides of fourteen protein coding genes using maximum likelihood (ML). The numbers behind each node denote the bootstrap support values. The bold indicates the species: *Sinularia acuta* that we sequenced its mitogenome in this paper. The following sequences were used: *Sinularia maxima* MN485891 (Chen et al. 2019), *Sinularia penghuensis* MW256412 (Shen et al. 2021), *Sinularia* acuta MW987591 (this study), *Sinularia* cf. *cruciata* NC_034318 (Shimpi et al. 2017), *Sinularia ceramensis* NC_044122 (Asem et al. 2019), *Sinularia peculiaris* NC_018379 (Kayal et al. 2015), and *Sarcophyton trocheliophorum* MK994517 (Shen et al. 2019).

For the first time, in the present study, we reported the complete mitochondrial genome mitogenomes of *Sinularia acuta* which has been successfully sequenced, assembled, and annotated. The constructed phylogenetic tree revealed that *Sinularia penghuensis*, *Sinularia maxima*, and *S. acuta* were located in the same clade, while *Sinularia ceramensis* and *Sinularia peculiaris* were clustered together in another clade (Figure 3).

## Conclusion

Generally, taxonomy and phylogenetic of genus *Sinularia* are problematic. We reported the first mitogenomes assembly and annotation of *Sinularia acuta*. The complete mitochondrial genome presented here could be utilized as a genomic data for further studies on phylogenetic analysis, evolutionary biology, and population genetics.

## Supplementary Material

Supplemental Material

## Data Availability

The mitogenome sequence data that support the findings of this study are openly available in GenBank of NCBI at https://www.ncbi.nlm.nih.gov/ under the accession no. MW987591. The associated Bio-Sample, BioProject, and SRA numbers are SAMN23175316, PRJNA780668, and SRR16961931, respectively.
